# Exploration of the cutoff values of axial length that is susceptible to develop advanced primary open angle glaucoma in patients aged less than 50 years

**DOI:** 10.1007/s00417-025-06827-4

**Published:** 2025-04-14

**Authors:** Kenji Suda, Masahiro Miyake, Tadamichi Akagi, Hanako Ohashi Ikeda, Takanori Kameda, Tomoko Hasegawa, Shogo Numa, Akitaka Tsujikawa

**Affiliations:** 1 Department of Ophthalmology and Visual Sciences, Kyoto University Graduate School of Medicine, 54 Kawahara-Cho, Shogoin, Sakyo-Ku, Kyoto, 606 - 8507 Japan; 2 Division of Ophthalmology and Visual Science, Graduate School of Medical and Dental Sciences, Niigata University, 1 - 754, Asahimachi-Dori, Chuo-Ku, Niigata, 951 - 8510 Japan

**Keywords:** Primary open-angle glaucoma, Myopia, Visual field, Axial length

## Abstract

**Purpose:**

To examine the correlation of the spherical equivalent refraction (SER) and axial length with the visual field, and to determine cutoff values of SER and axial length for the risk of developing advanced primary open-angle glaucoma (POAG).

**Methods:**

Patients with POAG or secondary glaucoma were retrospectively enrolled from a clinical database at Kyoto University Hospital between August 2011 and December 2018. Correlations between the mean deviation (MD) and axial length were evaluated in these patients using the Pearson’s correlation coefficient. Advanced POAG was defined as having an MD of less than –12 dB as measured by the Humphrey visual field analyzer (HFA). The cutoff values of the axial length and SER for the risk of developing advanced POAG were determined using Fisher’s exact test and Youden’s J statistic for HFA 24–2 and 10–2, respectively.

**Results:**

This study included 741 eyes of 438 patients. In POAG, axial length was negatively correlated with the MDs of the HFA 24–2 and 10–2. However, in secondary glaucoma, axial length did not significantly correlate with the MDs of the HFA. The cutoff axial length for the HFA 24–2 was 27.7 mm. For HFA 10–2, two peaks for the Youden’s J statistic were observed, at 27.67 mm and 25.51 mm. No significant association was found between SER and severity of visual field changes.

**Conclusions:**

Axial length measurement was clinically significant in assessing risk of severe or central visual field defects in young patients with POAG.

**Supplementary Information:**

The online version contains supplementary material available at 10.1007/s00417-025-06827-4.

## Abstract (highlights)

**Key messages:**

*****What is known***:**

- Spherical equivalent refraction (SER) and axial length (AL) are commonly used indices to evaluate myopia as a risk factor of primary open-angle glaucoma (POAG).
- The cutoff values of SER and AL are yet to be established as the best indicators for the onset of glaucoma.

*****What is new***:**

- The cutoff values of the AL for the risk of developing severe central visual field loss due to POAG were 27.67 mm and 25.51 mm.
- No significant association was found between SER and severity of visual field changes.
- The correlation between AL and severity of visual field in patients with POAG indicated that myopia may influence the onset of this disease in young patients.

**Supplementary Information:**

The online version contains supplementary material available at 10.1007/s00417-025-06827-4.

## Introduction

The prevalence of myopia has increased worldwide [1, 2]. Myopia tends to progress during school years, particularly in Asia, [3] and is a known risk factor for glaucoma, retinal detachment, macular degeneration, retinochoroidal atrophy, and cataracts. According to a meta-analysis, individuals with myopia (–3 D or less) have 2.5 times higher risk of developing glaucoma [4, 5]. Reports of some myopic cases indicate that changes in the optic disc have occurred since childhood, raising concerns about the risk of developing glaucoma at a young age [6]. Myopia and normal tension glaucoma (NTG) are prevalent in East Asia [7, 8], suggesting that myopia is an intraocular pressure (IOP)-independent risk factor for NTG. Jonas et al. reported that glaucomatous optic neuropathy in highly myopic eyes was not associated with IOP but rather with structural changes of optic nerve head characteristic of high myopia [9, 10]. However, there is still a need to clarify the relationship of myopia and NTG, as well as glaucoma with high IOP, particularly in conditions such as secondary glaucoma (SG).

Glaucomatous patients with myopia are more likely to have impaired paracentral visual field compared to those with primary open-angle glaucoma (POAG) without myopia.[11, 12]Spherical equivalent refraction (SER) and axial length (AL) are commonly used to evaluate myopia as a risk factor in epidemiological studies. While previous epidemiological studies have reported an association between myopia and glaucoma, most of these studies used SER as an indicator with defined cutoff values [4, 13, 14]. However, refractive surgery and cataract surgery can change the SER, making it difficult to use them as reliable indicators, particularly in older adults. AL is a more reliable indicator for adult cases, as it does not change after refractive or cataract surgery. However, it is not widely measured in general ophthalmological practice, except during preoperative examinations for cataract surgery. When used as an indicator of myopia, the cutoff value for AL is often set at 26.5 mm [15, 16] or 24 mm [17, 18]. Miyake et al. proposed that the onset of various myopia-related pathologies can be predicted from the radius of the curvature of the staphyloma, and different cutoff values can be set according to the type of myopia complications [19]. However, the cutoff values for SER and AL are yet to be established as the best indicators for the onset of glaucoma.

We conducted a retrospective epidemiological study on young-onset glaucoma (age < 50 years), which is less influenced by aging and cataract surgery. We aimed to clarify the differences between POAG and SG regarding myopia and visual field impairment, verify the correlation between SER or AL and visual field changes, and determine the appropriate cutoff values for SER and AL in assessing the risk of advanced POAG.

## Materials and methods

This cohort study adhered to the tenets of the Declaration of Helsinki and was approved by the Institutional Review Board and Ethics Committee of Kyoto University Graduate School of Medicine (approval number [R2652]). The requirement for written informed consent was waived due to the retrospective design of this study. Instead, we publicly disclosed results of this retrospective research on our faculty's website, while allowing participants to opt-out of the study. Under this condition, the Institutional Review Board approved the waiver for informed consent.

## Subjects

Subjects were retrospectively enrolled at the Kyoto University Hospital from a clinical database of patients who visited our glaucoma clinic between August 2011 and December 2018. Patients with glaucoma that were < 50 years on the day of their first visual field test were included in this study. Patients diagnosed with preperimetric glaucoma (PPG), primary angle-closure glaucoma, suspected optic disc hypoplasia, or no glaucomatous optic neuropathy (GON) were excluded. Ultimately, we included the eyes with uveitis, steroid-induced glaucoma, neovascular glaucoma, and developmental glaucoma as SG in this study and compared their characteristics with those of POAG, most of which consist of NTG. Patients with other ocular disorders, such as diabetic retinopathy, age-related macular degeneration, or intracranial diseases affecting visual function, were also excluded. If the age at the first Humphrey visual field analyzer (HFA; Carl Zeiss-Meditec, Dublin, CA, USA) 10–2 Swedish Interactive Threshold Algorithm (SITA) standard protocol was > 50 years, the result of the HFA 10–2 was excluded from the analyses.

## Data collection

Subjects’ clinical characteristics were extracted from clinical records, including type of glaucoma, age at the first visual field examination using the HFA 24–2 and 10–2 SITA standard testing protocol, first mean deviations (MDs) of the HFA 24–2 and 10–2, refractory power (ARK- 530 A; Nidek, Gamagori, Japan) and IOP measured using a Goldmann applanation tonometer at the first visit, AL (IOLMaster 500; Carl Zeiss Meditec, Dublin, California, USA), and central corneal thickness (SP- 3000; Tomey, Tokyo, Japan). The reliability of the visual field tests was defined using fixation loss (< 20%), false-positive error rate, and false-negative rate (< 33%). Only reliable tests were used for the analysis.

## Statistical analysis

Fisher’s exact test was used to compare categorical values. Correlations between the MD and AL were evaluated in patients with POAG or SG using the Pearson’s correlation coefficient. To compare the means and evaluate the effects influencing MDs, we used a generalized estimating equation (GEE) for the univariable and multivariable regression analyses. In the GEE framework, each eye is considered individually dependent. GEE analyses were performed using the R package ‘geepack’.

To determine the cutoff values of AL and SER for developing advanced POAG in young patients, the Youden index was used. Advanced glaucoma was defined as MD under –12 dB in the HFA according to the Anderson’s criteria. We explored the cutoff values of AL and SER for advanced POAG based on the Fisher’s exact test, and determined the values with the highest Youden’s J statistic for the HFA 24–2 and 10–2, respectively. Youden’s J statistic was calculated using sensitivity + specificity – 1. To calculate the Youden’s index and draw the receiver operating characteristic (ROC) curves, we used the ROCR package in R.

We performed intraclass correlation coefficient (ICC) test to determine whether both eyes could be included or not [20, 21]. We evaluated the consistency of MD of the right and left eyes in patients whose both eyes met the inclusion criteria. ICCs were calculated in MDs of HFA 24–2 and 10–2 using the ‘irr’ package in R.

All p-values analyzed are two-sided. Statistical significance was defined as p < 0.05. All analyses were performed using the R ver. 3.6.0 software (R Foundation for Statistical Computing, Vienna, Austria).

## Results

A total of 741 eyes of 438 patients (229 female, 209 male) were included in this study. A description and comparison of the clinical characteristics of the subjects with POAG and SG are shown in Table 1. ICCs of MDs were 0.569 in HFA 24–2 and 0.649 in HFA 10–2; therefore, both eyes of all patients were included in the following analyses.

**Table 1:** Clinical characteristics of the subjects

	Total	POAG	Secondary glaucoma	*p* value
Patients	438	324	116	
Eyes	741	548	193	
Gender (female/male)	229/209	159/165	51/65	0.28
Intraocular pressure at first visit (mmHg)	**16.9 ± 6.3**	**16.0 ± 3.8**	**19.5 ± 10.1**	** < 0.001**
Spherical equivalent refraction at first visit (D)	**‒6.57 ± 4.57**	**‒7.22 ± 4.18**	**‒4.70 ± 5.08**	** < 0.001**
MD of HFA 10–2 at first visit (dB)	**‒11.7 ± 9.6**	**− 10.1 ± 8.8**	**‒18.7 ± 10.2**	** < 0.001**
Age at first experiment of HFA 10–2 (year)	**42.1 ± 8.8**	**43.1 ± 8.2**	**37.4 ± 9.9**	**0.001**
MD of HFA 24–2 at first visit (dB)	**‒9.5 ± 8.3**	**‒8.6 ± 7.4**	**‒12.0 ± 10.2**	** < 0.001**
Age at first experiment of HFA 24–2 (year)	**38.7 ± 8.7**	**40.1 ± 7.7**	**34.8 ± 10.1**	** < 0.001**
Central corneal thickness (µm)	528 ± 34.9	528 ± 34.3	530 ± 37.1	0.70
Axial length (mm)	**26.5 ± 1.9**	**26.7 ± 1.8**	**25.7 ± 1.9**	** < 0.001**
MD of HFA 24–2 at final visit (dB)	**‒11.0 ± 8.6**	**‒10.1 ± 7.6**	**‒13.7 ± 10.7**	** < 0.001**
Rate of change in HFA 24–2 (dB/year)	**‒**0.34 ± 1.39	**‒**0.32 ± 0.91	**‒**0.43 ± 2.40	0.63
Experiment times of HFA 24–2	**10.1 ± 8.5**	**11.5 ± 8.8**	**6.0 ± 5.7**	** < 0.001**
Best corrected visual acuity (logMAR)	**‒0.006 ± 0.33**	**‒0.05 ± 0.27**	**0.09 ± 0.44**	** < 0.001**

Values are shown as mean ± standard deviation. Statistically significant values are shown in bold

Comparisons were performed using the unpaired t-test (continuous value) or Fisher’s exact test (categorical value)

*POAG* Primary open-angle glaucoma, *MD* Mean deviation, *HFA* Humphrey field analyzer

Although the average age of the SG group was lower than that of the POAG group, the MDs of HFA 24–2 and 10–2 were more severe. In terms of SER and AL, eyes with POAG were more myopic than those with SG. The detailed characteristics of participants are documented in Supplementary Table S1.

**Fig. 1:**
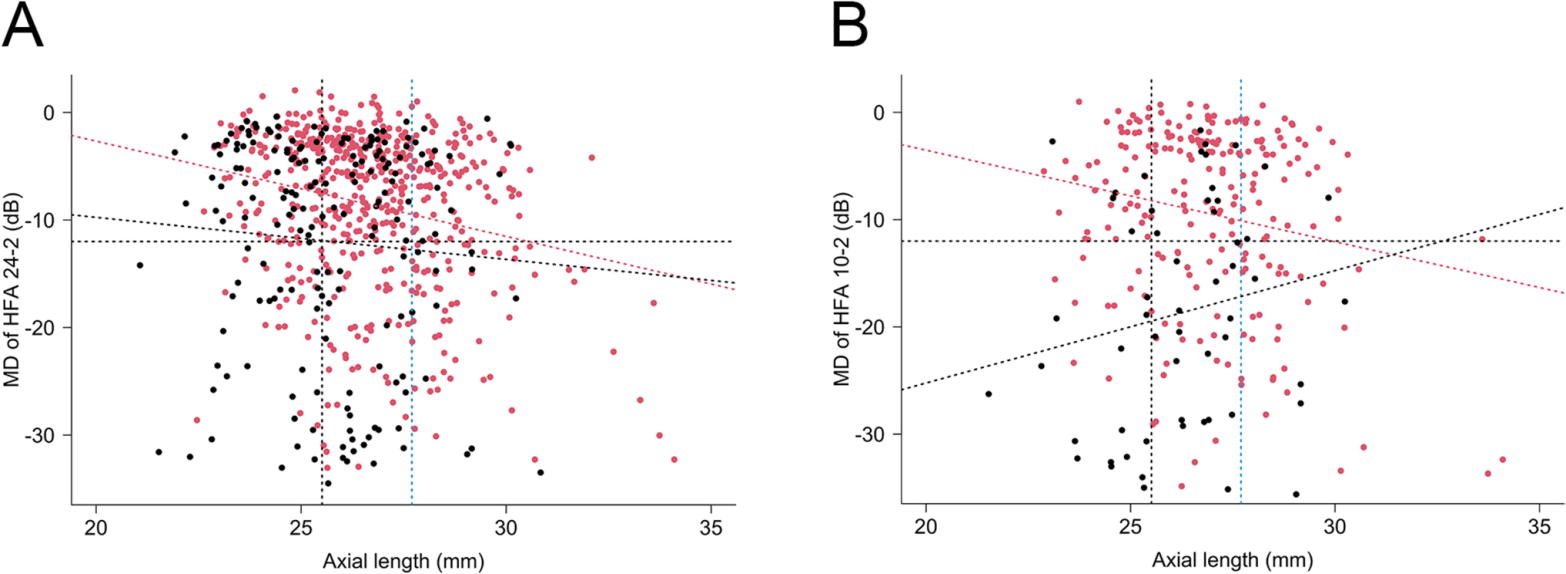
Scatter plots showing the correlation between the axial length and MD of the HFA (**A**) 24–2 and (**B**) 10–2 protocols. Each dot represents one eye. Red spots indicate POAG and black spots indicate secondary glaucoma. In POAG, axial length was negatively correlated with both MD of the HFA 24–2 (R = − 0.22, *p* < 0.0001) and 10–2 (R = − 0.16, *p* = 0.007) protocols (red dotted lines). However, in secondary glaucoma, axial length was not correlated with either the MD of the HFA 24–2 (R = − 0.07, *p* = 0.32) or 10–2 (R = 0.10, p = 0.45) protocol (black oblique lines). The two vertical lines indicate the cutoff values of the axial length. The black vertical line is 25.5 mm and the blue line is 27.7 mm. The black horizontal line indicates − 12 dB of MD of the HFA. MD, mean deviation; HFA, Humphrey visual field analyzer; POAG, primary open-angle glaucoma

The correlations between the AL and MDs of the HFA are shown in Fig. 1. In POAG, AL was negatively correlated with both MDs of the HFA 24–2 (R = − 0.22, 95% confidence interval [CI]: –0.30 to –0.14, *p* < 0.0001) and 10–2 (R = − 0.16, 95% CI: –0.28 to –0.04, p = 0.007) protocol; however, in SG, the AL was not significantly correlated with MDs of the HFA 24–2 (R = –0.07, 95% CI: –0.21 to 0.07, *p* = 0.32) and 10–2 (R = 0.10, 95% CI: –0.16 to 0.34, *p* = 0.45) protocols.

**Fig. 2:**
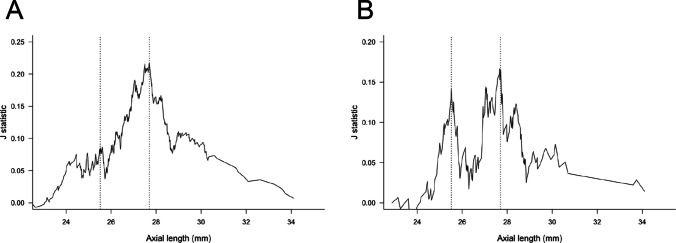
Exploration of the cutoff values of the axial length by Youden J statistic. The line plots show changes in the Youden’s J statistic with axial length. When the Youden index was calculated, the relationship between the axial length and advanced primary open-angle glaucoma (defined as less than − 12 dB of mean deviation of the HFA) was evaluated using the Fisher’s exact test. **A** In the HFA 24–2 protocol, the line plot of the J statistic shows only one peak. **B** In the HFA 10–2, the line plot of the J statistic shows two peaks. The two vertical lines indicate the cutoff values of the axial length. The black dotted line is 25.5 mm and the blue dotted line is 27.7 mm. HFA, Humphrey visual field analyzer

To explore the cutoff values of AL for developing advanced POAG in young patients, we used the Youden index on the AL and MDs of the HFA 24–2 or 10–2 (Fig. 2). When advanced POAG was defined by MD under − 12 dB in the HFA 24–2, the cutoff value of AL was 27.7 mm (Youden’s J statistic: 0.22, sensitivity: 0.44, specificity: 0.77; *p* < 0.001 in the Fisher’s exact test; Fig. 2A). For HFA 10–2, the Youden’s J statistic showed two peaks (Fig. 2B), which were 27.67 mm (Youden’s J statistic: 0.16, sensitivity: 0.46, specificity: 0.70; *p* = 0.02 in the Fisher’s exact test) and 25.51 mm (Youden’s J statistic: 0.16, sensitivity: 0.85, specificity: 0.28; *p* = 0.03 in the Fisher’s exact test). The ROC curves showing the diagnostic ability of AL or SER for advanced glaucoma defined by HFA 24–2 or 10–2 are depicted in Supplementary Figure S1.

**Fig. 3:**
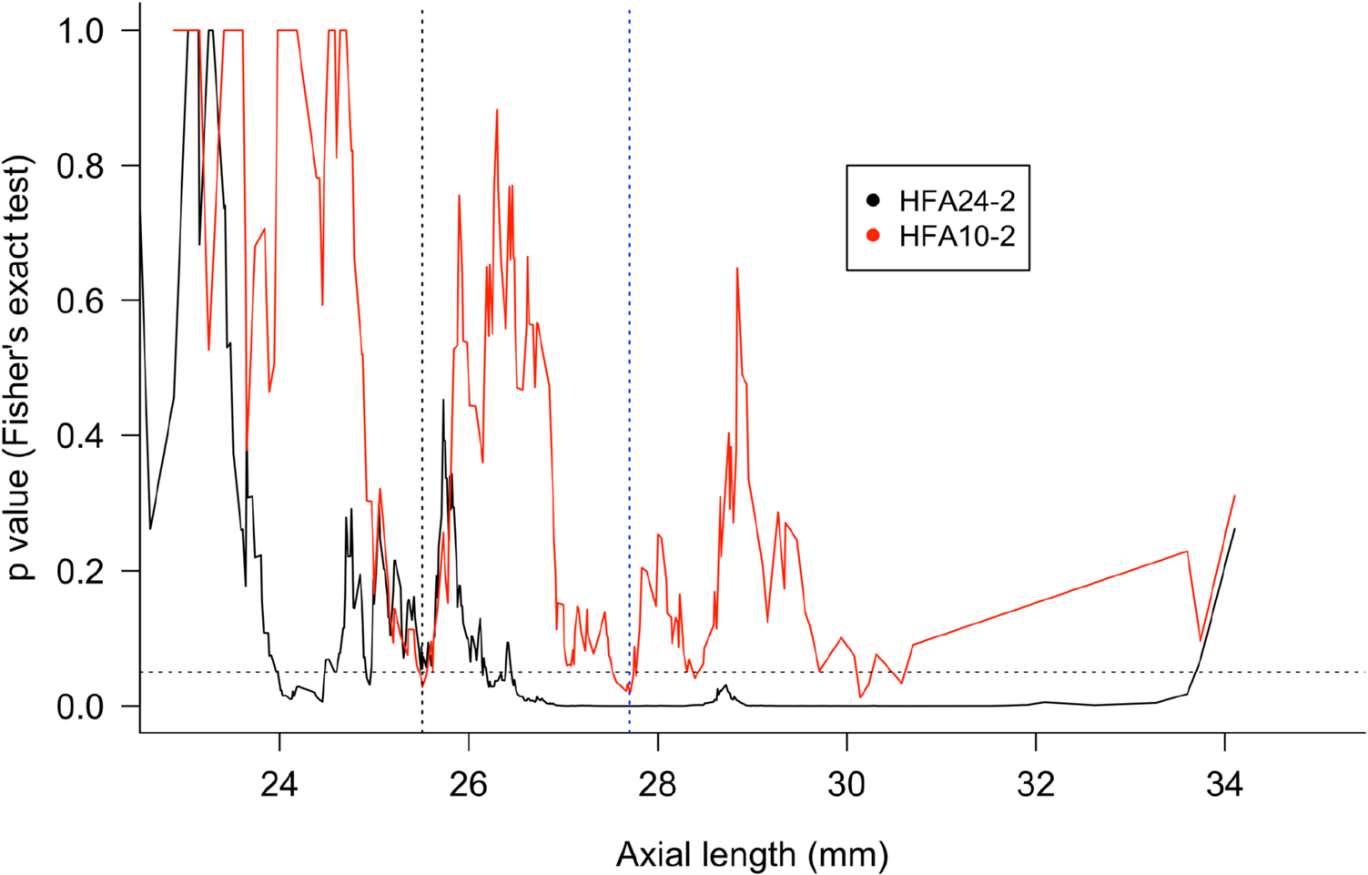
Changes in the p-value of the Fisher’s exact tests depending on the cutoff values of axial length for advanced POAG. The Fisher’s exact tests evaluated the relationship between the cutoff values of axial length and advanced POAG (defined as less than − 12 dB of mean deviation of the HFA). The line plots show changes in the p-values of the Fisher’s exact tests according to the cutoff value for axial length. The black and red lines indicate the results of the HFA 24–2 and 10–2 protocols, respectively. The two vertical lines indicate the cutoff values of axial length. The black dotted line is 25.5 mm and the blue dotted line is 27.7 mm. The horizontal line indicates a *p*-value of 0.05. HFA, Humphrey visual field analyzer; POAG, primary open-angle glaucoma.

Changes in p-values in the Fisher’s exact tests when the cutoff value for AL varied are shown in Fig. 3. The p-values of the AL at 25.51 mm and 27.7 mm in Fisher’s exact tests were smaller than other cutoff values for AL in the HFA 10–2. When the AL was set to 25.51 mm, the p-value of the Fisher’s exact test in the HFA 24–2 was 0.013 (Youden’s J statistic: 0.11, sensitivity: 0.81, specificity: 0.30). In Table 2, the clinical characteristics of POAG were compared between the groups divided using the cutoff value for AL determined by Youden’s index (25.5 mm and 27.7 mm). In myopic patients, the proportions of female and younger patients were significantly higher. The MDs of the HFA 24–2 and 10–2 were significantly lower in the myopic groups, but the differences in MDs of the HFA 10–2 were marginally significant when the groups were divided according to both cutoff values. A comparison of clinical characteristics of patients with SG is presented in Supplementary Table S2.

**Table 2:** Comparison of the clinical characteristics of patients with POAG between the groups divided by the cutoff value of axial length determined by the Youden’s index

	AL < 27.7 mm	AL > = 27.7 mm	*P* value	AL < 25.5 mm	AL > = 25.5 mm	*P* value
Patients	240	99		87	250	
Eyes	394	154		135	413	
Gender (female/male)	**133/107**	**30/69**	** < 0.001**	**54/33**	**112/138**	**0.006**
Intraocular pressure at first visit (mmHg)	15.9 ± 3.8	16.2 ± 3.7	0.45	16.1 ± 4.1	15.9 ± 3.6	0.69
Spherical equivalent refraction at first visit (D)	**− 5.65 ± 3.04**	**− 11.20 ± 4.02**	** < 0.001**	**− 3.05 ± 2.40**	**− 8.59 ± 3.71**	** < 0.001**
Best corrected visual acuity at first visit at first visit (logMAR)	**− 0.074 ± 0.23**	**0.019 ± 0.32**	**0.01**	**− 0.089 ± 0.18**	**− 0.032 ± 0.29**	**0.025**
MD of HFA 10–2 at first visit (dB)	− 9.40 ± 8.40	− 11.80 ± 9.40	0.075	− 8.54 ± 7.39	− 10.70 ± 9.15	0.088
Age at first experiment of HFA 10–2 (year)	43.7 ± 8.4	41.5 ± 7.5	0.10	**45.3 ± 7.6**	**42.3 ± 8.3**	**0.027**
MD of HFA 24–2 at first visit (dB)	**− 7.69 ± 6.95**	**− 11.0 ± 7.90**	** < 0.001**	**− 6.97 ± 6.49**	**− 9.17 ± 7.57**	**0.006**
Age at first experiment of HFA 24–2 (year)	40.4 ± 7.9	39.2 ± 7.1	0.18	41.2 ± 7.3	39.7 ± 7.8	0.11
Central corneal thickness (µm)	526 ± 35	534 ± 31	0.10	528 ± 34	528 ± 34	1.00
Axial length (mm)	**25.9 ± 1.2**	**29.0 ± 1.2**	** < 0.001**	**24.5 ± 0.7**	**27.4 ± 1.5**	** < 0.001**
MD of HFA 24–2 at final visit (dB)	**− 9.18 ± 7.16**	**− 12.50 ± 8.16**	** < 0.001**	**− 8.35 ± 6.76**	**− 10.70 ± 7.77**	**0.006**
Rate of change in HFA 24–2 (dB/year)	− 0.31 ± 0.97	− 0.35 ± 0.74	0.70	− 0.27 ± 1.33	− 0.34 ± 0.71	0.65
Experiment times of HFA 24–2	11.7 ± 8.67	10.8 ± 9.24	0.41	**13.2 ± 8.87**	**10.9 ± 8.76**	**0.047**

Values are shown as mean ± standard deviation. Statistically significant values are shown in bold

Comparisons were performed using the unpaired t-test (continuous value) or Fisher’s exact test (categorical value)

*AL* Axial length, *MD* Mean deviation, *HFA* Humphrey field analyzer

The relationship between the SER and MD of the HFA is shown in Supplementary Figure S2. In POAG, refraction was correlated with the MD of the HFA 24–2 (R = 0.18, 95% CI: –0.096 to 0.26, *p* < 0.001) but not with that of HFA 10–2 (R = 0.44, 95% CI: –0.06 to 0.22, *p* = 0.28). We also explored the cutoff value of the SER in the same manner, which is presented in Supplementary Figure S3. The largest Youden’s J statistic was − 4.125 D in HFA 24–2 and − 6.875 D in HFA 10–2. However, for both cutoff values, the Fisher’s exact test revealed no significant association between refraction and visual field severity.

Table 3 shows the univariable and multivariable regression for evaluation of the factors influencing the MDs of the HFA 24–2 and 10–2 at the first visit in patients with POAG. In the multivariable regression, SER was excluded from the explanatory variables because the correlation of AL with SER was high (R = − 0.80). After adjustment for sex, age at first visit, IOP at first visit, central corneal thickness, and AL, the AL was significantly correlated with the MD of the HFA 24–2 (β=–0.844 ± 0.377, 95% CI: –1.58 to –0.104, *p* = 0.025) but not with the MD of the HFA 10–2 (β=–0.352 ± 0.457, 95% CI: –1.25 to 0.544, *p* = 0.44).

**Table 3:** Univariable and multivariable regression analysis using the general estimating equation to evaluate the factors influencing the visual fields of patients with POAG

	Univariable Regression	Multivariable Regression*
	β (95% CI), *p* value	
Variable		
MD of HFA 24–2 at first visit		
Gender (female = 1)	**1.92 ± 0.93 (0.10 to 3.74),* p* = 0.039**	1.12 ± 1.31 (− 1.45 to 3.69),* p* = 0.39
Age at first visit (year)	‒0.11 ± 0.06 (‒0.22 to 0.009), *p* = 0.070	**− 0.18 ± 0.084 (− 0.34 to − 0.010),* p* = 0.038**
IOP at first visit (mmHg)	0.10** ± **0.14 (‒0.17 to 0.38), *p* = 0.46	0.22 ± 0.15 (− 0.080 to 0.52),* p* = 0.15
Central corneal thickness (µm)	‒0.003 ± 0.016 (− 0.034 to 0.028), *p* = 0.85	‒0.013 ± 0.022 (‒0.055 to 0.030),* p* = 0.56
Axial length (mm)	‒**0.86 ± 0.28 (**‒**1.4 to **‒**0.32), *p* = 0.002**	‒**0.84 ± 0.38 (**‒**1.58 to − 0.10),* p* = 0.025**
Spherical equivalent refraction at first visit (D)	**0.38 ± 0.11 (0.16 to 0.60),* p* < 0.001**	-
MD of HFA 10–2 at first visit		
Gender (female = 1)	**2.93 ± 1.43 (0.13 to 5.74),* p* = 0.041**	**3.60 ± 1.77 (0.13 to 7.07),* p* = 0.042**
Age at first visit (year)	‒0.14 ± 0.09 (− 0.31 to 0.043),* p* = 0.14	‒0.16 ± 0.12 (− 0.40 to 0.084), *p* = 0.2
IOP at first visit (mmHg)	0.36 ± 0.21 (‒0.05 to 0.78), *p* = 0.088	**0.62 ± 0.22 (0.19 to 1.06),* p* = 0.005**
Central corneal thickness (µm)	‒0.017** ± **0.021 (− 0.056 to 0.025), *p* = 0.42	− 0.032 ± 0.023 (− 0.078 to 0.013), *p* = 0.17
Axial length (mm)	‒0.69 ± 0.37 (‒1.42 to 0.049), *p* = 0.067	‒0.35 ± 0.46 (‒1.25 -to 0.54),* p* = 0.44
Spherical equivalent refraction at first visit (D)	0.18 ± 0.16 (‒0.12 to 0.48), *p* = 0.25	-

*MD* Mean deviation, *HFA* Humphrey field analyzer, *IOP* Intraocular pressure

*Adjusted by gender, age at first visit, IOP at first visit, central corneal thickness and axial length

## Discussion

The correlation between AL and the severity of the visual field in patients with POAG indicated that myopia may influence the onset of POAG in young patients, while high IOP plays a major role in the development of SG [7, 8]. Our study revealed that only AL, not SER, was correlated with the MD of the HFA in patients with POAG. The pathological changes in the optic disc in myopic glaucoma are known to be different from those in glaucoma without myopia, such as intrachoroidal cavitation [22], focal lamina cribrosa defects [23], gamma-zone PPA, [24, 25] or adduction-induced strain [26]. These studies indicate that GON with myopia has other pathological mechanisms induced by axial elongation of the eyeball.

The analyses using the Youden index proposed two cut off values for AL, namely 27.7 mm and 25.5 mm. Previous clinical studies on glaucoma conventionally adopted 26.5 mm or 24 mm as the cutoff value. To the best of our knowledge, only the study by Jonas et al. set 27.5 mm as the cutoff value of AL for dividing GON in terms of IOP dependency [9]. When the AL is > 27.5 mm, GON only correlated with older age, shorter vertical diameter of the temporal arterial arcade, and longer minimal optic disc diameter, in addition to AL. A study by Flores-Morteno used the cutoff value of 28 mm for pathological myopia, which was observed using multimodal imaging [27]. These arguments suggest that eyes with ALs > 28 mm should be excluded from clinical studies on GON.

The results of the current study showed that another cutoff value of AL for the severity of the central visual field in young patients with POAG was 25.5 mm. Similar to 27.7 mm, there are a few reports wherein the cutoff value was set to 25 mm. Melo et al. reported a short letter regarding the evaluation of GON with high myopia, which was defined by an AL of 25 mm with multimodal imaging [28], but they did not discuss why this value was chosen. Moreover, they failed to discriminate highly myopic GON using imaging technologies; thus, the significance of this definition remains unclear. Another observational study adopting the same definition of myopia focused on the diagnostic ability of optical coherence tomography parameters for myopic PPG [29]. They revealed that parameters calculated from the ganglion cell complex thickness had the best diagnostic performance compared to those calculated from the retinal nerve fiber layer (RNFL) thickness; however, the diagnostic performance was similar regardless of whether the AL was more or less than 25 mm. Hence, no clinical reports on glaucoma have discussed the significance of 25 mm or 25.5 mm as the cutoff value of AL.

Discussion of 25 mm AL as a cutoff value has arisen from myopic maculopathy. According to the International Photographic Classification and Grading System for Myopic Maculopathy, it is defined as the presence of one or more lesions caused by myopic changes, such as diffuse chorioretinal atrophy at the posterior pole, patchy chorioretinal atrophy, macular atrophy, or additional lesions (lacquer cracks, Fuchs spots, or myopic choroidal neovascularization) [30]. A report from the Hisayama study showed that the optimal cutoff values of AL for myopic maculopathy were 25.9 mm in men and 25.3 mm in women [31]. In the current study, participants were diagnosed with POAG or SG; however, visual field damage within the central 10° in myopic eyes was not necessarily derived from glaucoma, but from myopic changes, especially myopic maculopathy. Although we did not analyze the clinical data of structural changes or the relationship between structure and function, this investigation of macular function approached by both structure and function may elucidate a new aspect of central visual field loss in myopic glaucoma. For example, Koh et al. reported that subfoveal choroidal thickness was associated with central visual field defects rather than AL or age, which are considered as risk factors for glaucoma [32].

This study has some limitations. First, it had a retrospective design, and the history of medical or surgical treatment was not investigated in detail; however, the subjects were young (< 50 years) and most patients with POAG were not treated aggressively. Second, all participants were Japanese (Asians). The AL results were not applied directly to other races because of physical differences. Similarly, height and weight may need to be considered when analyzing the AL, even in the same race. Finally, the cause of visual field defects in this study may not be GON. We recruited subjects from the glaucoma clinic in our hospital, where glaucoma experts diagnosed glaucomatous changes of the optic disc and corresponding visual field defects, but we could not exclude subjects with myopic maculopathy since maculopathy can coexist in eyes with an AL of 25 mm. Moreover, the subjects of this study also included eyes with myopic optic neuropathy. Further analyses, including function (visual field tests) and structure (e.g., optical coherence tomography), are needed.

In conclusion, in this retrospective hospital-based cohort of youth-onset POAG, we suggested two cutoff values for AL that might be able to differentiate optic neuropathy between glaucoma induced by high IOP and axial elongation of the eyeball. The measurement of AL in glaucoma management is clinically significant for estimating the risk of severe or central visual field defects in young individuals. Future studies are expected to reveal the correlation of these cutoff values with detailed pathological mechanisms that induce damage to the optic nerve accompanying axial elongation of the eyeball or the genetic background associated with GON.

## Supplementary Information

Below is the link to the electronic supplementary material.

Supplementary file1 (DOCX 652 KB)

## Data Availability

Data are available upon request.

## Abbreviations

AL: Axial length
CI: Confidence interval
GEE: Generalized estimating equation
GON: Glaucomatous optic neuropathy
HFA: Humphrey visual field analyzer
IOP: Intraocular pressure
MD: Mean deviation
POAG: Primary open-angle glaucoma
PPG: Preperimetric glaucoma
RNFL: Retinal nerve fiber layer
ROC: Receiver operating characteristic
SER: Spherical equivalent refraction
SG: Secondary glaucoma
SITA: Swedish Interactive Threshold Algorithm

## Declarations

### Provenance and peer review

Not commissioned; externally peer reviewed.
