# Supplementary material for: Exploration of the cutoff values of axial length that is susceptible to develop advanced primary open angle glaucoma in patients aged less than 50 years
Source: Graefes Arch Clin Exp Ophthalmol. 2025 Apr 14;263(9):2631–9. doi: 10.1007/s00417-025-06827-4 (PMC12513891; doi:10.1007/s00417-025-06827-4)
Supplement: Supplementary file 1 — Supplementary file1 (DOCX 652 KB) [file 417_2025_6827_MOESM1_ESM.docx]

**Exploration of the Cutoff Values of Axial Length that is Susceptible to Develop Advanced Primary Open Angle Glaucoma in Patients Aged Less than 50 years**

Kenji Suda, MD, PhD^1^**^*^**, Masahiro Miyake, MD, PhD^1^, Tadamichi Akagi MD, PhD^1,2^, Hanako Ohashi Ikeda, MD, PhD^1^, Takanori Kameda, MD, PhD^1^, Tomoko Hasegawa, MD, PhD^1^, Shogo Numa, MD, PhD^1^, Akitaka Tsujikawa, MD, PhD^1^

1, Department of Ophthalmology and Visual Sciences, Kyoto University Graduate School of Medicine, 54 Kawahara-cho, Shogoin, Sakyo-ku, Kyoto 606-8507, Japan

2, Division of Ophthalmology and Visual Science, Graduate School of Medical and Dental Sciences, Niigata University, 1-754, Asahimachi-dori, Chuo-ku, Niigata city, Niigata, 951-8510, Japan

**^*^Corresponding Author:** Kenji Suda, MD, PhD

Department of Ophthalmology and Visual Sciences

Kyoto University Graduate School of Medicine

54 Shougoin Kawahara-cho

Sakyo-ku, Kyoto 606-8507, Japan

Tel: +81-75-751-3248

Fax: +81-75-752-0933

Email: qwm04525@kuhp.kyoto-u.ac.jp

**
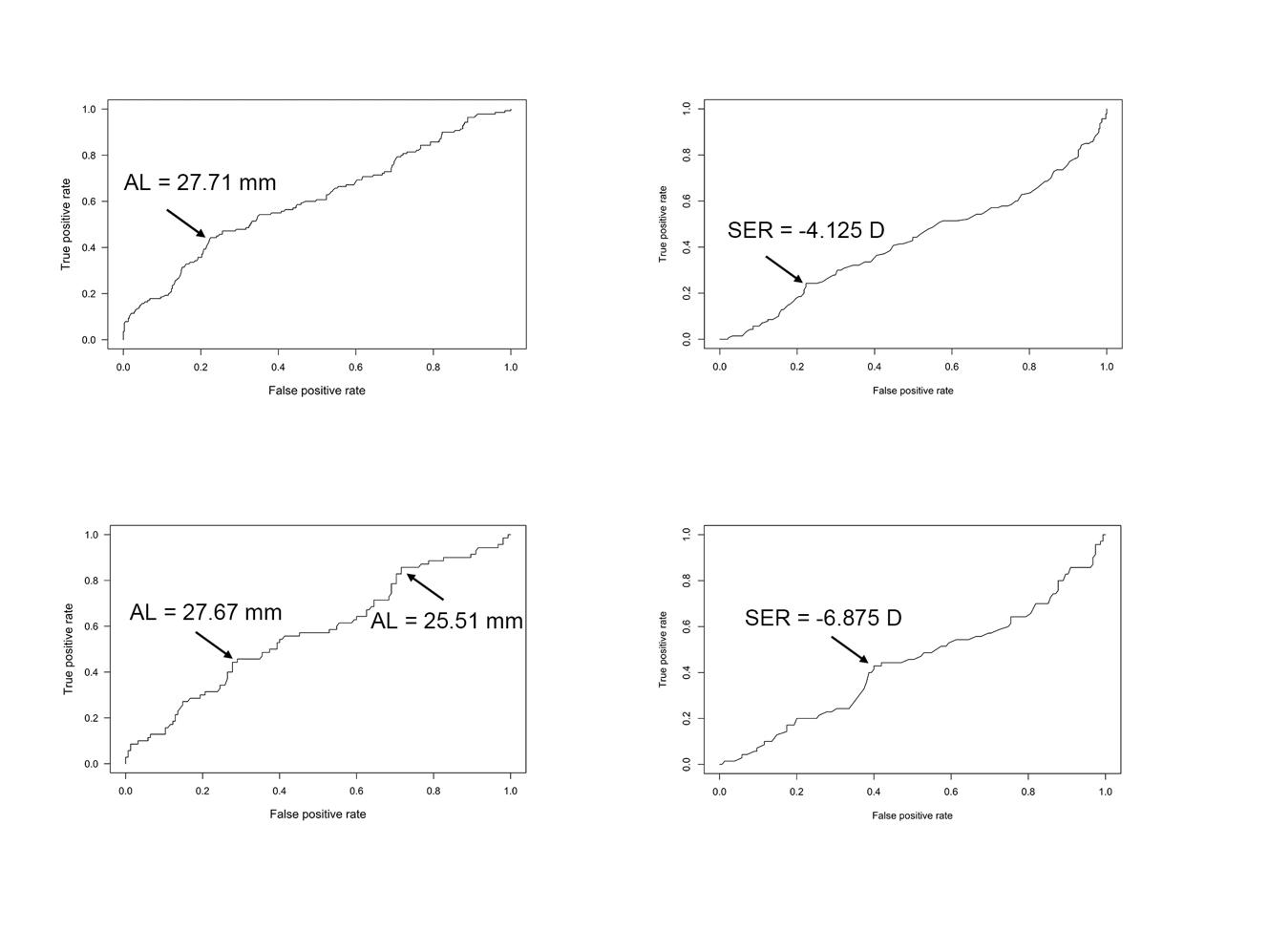
**

**Supplementary Figure S1.** Receiver operatorating characteristic (ROC) curves showing the diagnostic ability of axial length (AL) or spherical equivalent refraction (SER) for advanced primary open angle glaucoma (POAG) defined by MD under -12 dB in Humphrey visual field analyzer (HFA) using the 24-2 and 10-2 SITA standard testing protocol. Arrows in the figures show the cutoff values determined by Youden index. (A) AL in HFA 24-2. (B) AL in HFA 10-2. (C) SER in HFA 24-2. (D) SER in HFA 10-2.

**
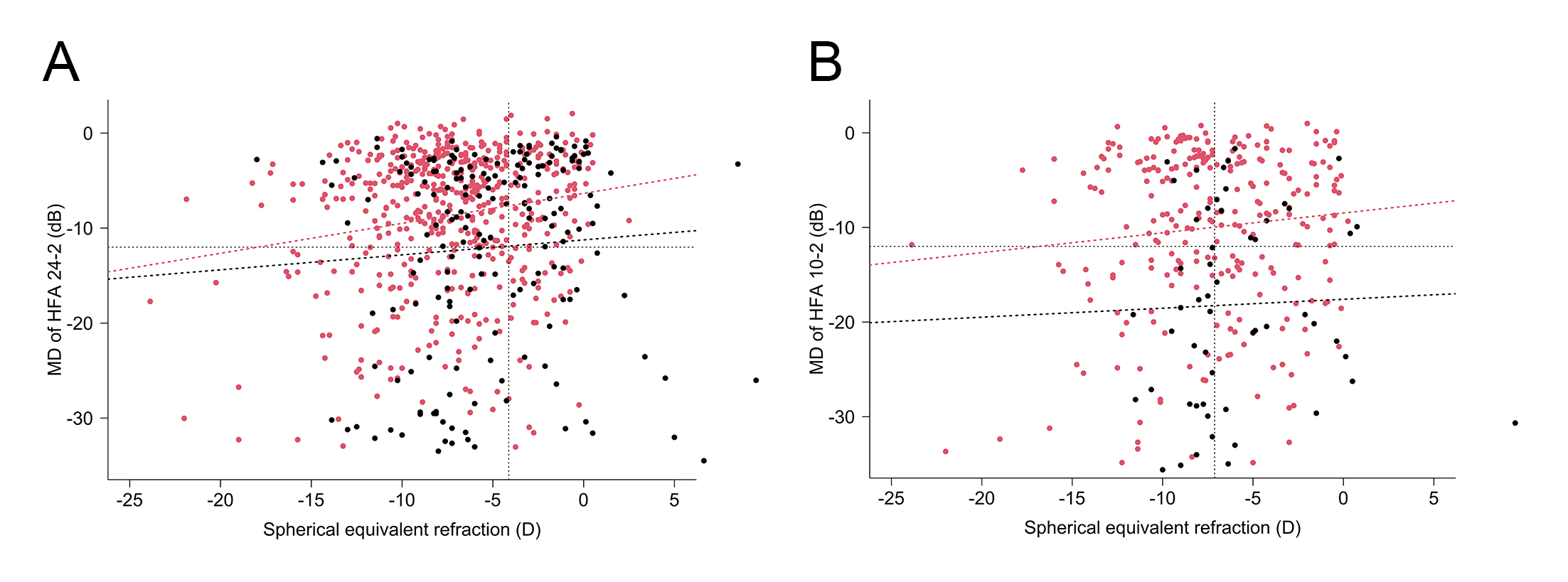
**

**Supplementary Figure S2.** Scatter plots showing the correlation between the spherical equivalent refraction and MD of the HFA (A) 24-2 and (B) 10-2 protocols. Each dot represents one eye. Red spots indicate POAG and black spots indicate secondary glaucoma. In POAG, refraction did not correlate with either the MD of the HFA 24-2 (R=0.085, p=0.11) or 10-2 (R=0.44, p=0.66) protocol (red dotted lines).

Similarly, in secondary glaucoma, refraction did not correlate with either the MD of the HFA 24-2 (R=0.11, p=0.20) or 10-2 (R=-0.02, p=0.90) protocol (black oblique lines). The black vertical lines correspond to (A) -4.125 D and (B) -7.125 D. MD, mean deviation; HFA, Humphrey visual field analyzer; POAG, primary open-angle glaucoma.

**
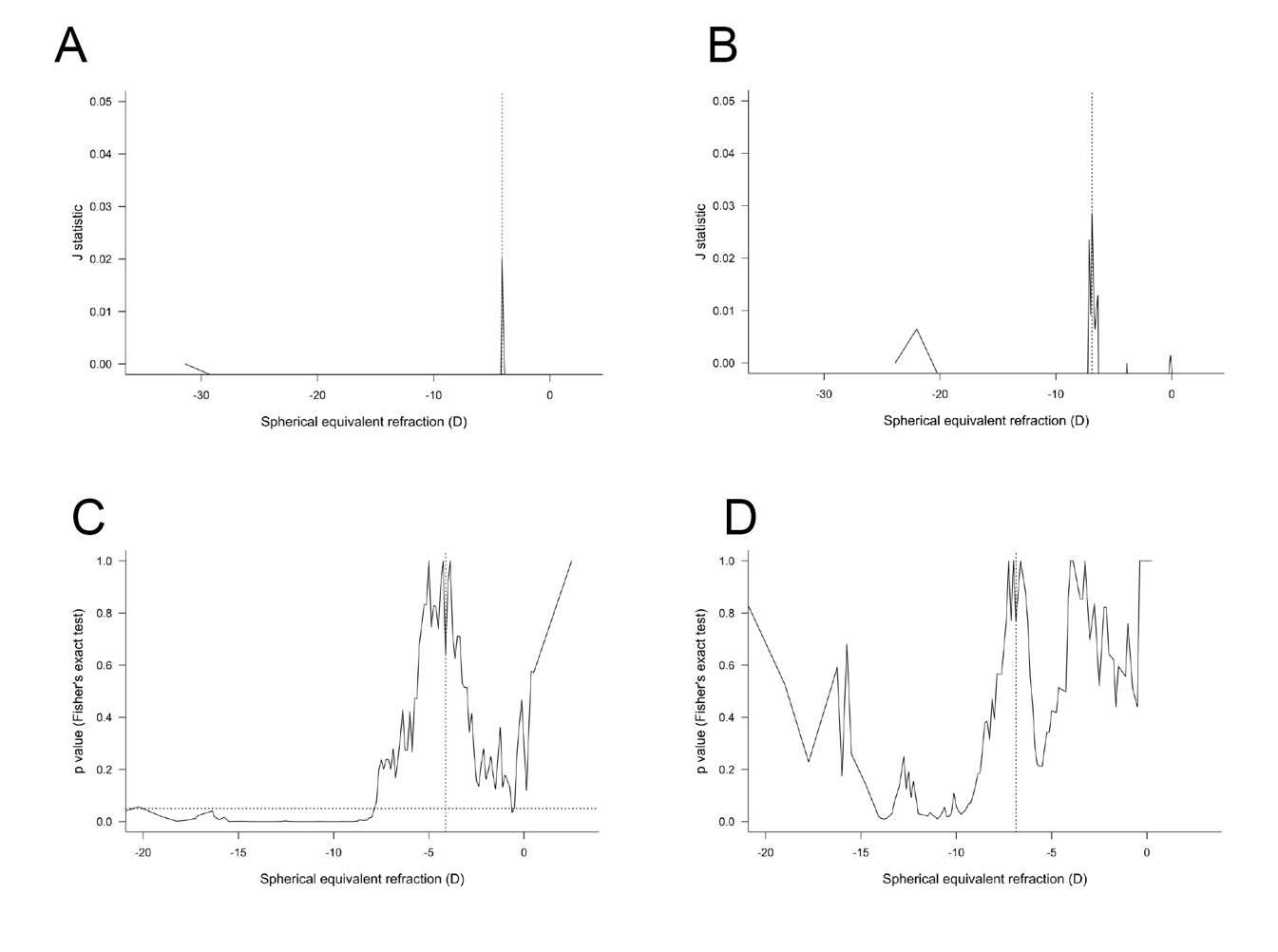
**

**Supplementary Figure S3.** Changes in the Youden’s J statistic and p-value of the Fisher’s exact tests depending on the cutoff values of the spherical equivalent refraction for advanced glaucoma. The Fisher’s exact tests evaluated the relationship between the cutoff values of axial length with advanced glaucoma (defined as less than -12 dB of mean deviation of the HFA). The highest peaks in the line plot of the Youden’s J statistic in the HFA (A) 24-2 and (B) 10-2 protocols were -4.125 D and -7.125 D spherical equivalent refraction, respectively. However, the Fisher’s exact tests revealed that the cutoff values of spherical equivalent refraction proposed by the Youden index did not show significant relationship with advanced glaucoma. HFA, Humphrey visual field analyzer.

**Supplementary Table S1** Clinical characteristics of the subjects with secondary glaucoma in the current study

|  | Uveitis | Steroid-induced | Neovascular | Developmental |
| --- | --- | --- | --- | --- |
| Patients | 29 | 51 | 5 | 25 |
| Eyes | 45 | 84 | 8 | 44 |
| Gender (female / male) | 13 / 16 | 25 / 26 | 1 / 4 | 11 / 14 |
| Intraocular pressure at first visit (mmHg) | 18.0 ± 9.3 | 18.8 ± 7.9 | 23.2 ± 23.4 | 20.8 ± 10.6 |
| Spherical equivalent refraction at first visit (D) | -3.5 ± 4.95 | -6.16 ± 3.33 | -0.969 ± 5.26 | -3.5 ± 6.73 |
| MD of HFA 10-2 at first visit (dB) | -16.5 ± 7.02 | -13.8 ± 9.29 | -27.9 ± 3.96 | -24.6 ± 9.20 |
| Age at first experiment of HFA 10-2 (year) | 46.1 ± 11.6 | 36.4 ± 7.7 | 38.0 ± 6.9 | 34.8 ± 8.0 |
| MD of HFA 24-2 at first visit (dB) | -10.6 ± 9.96 | -9.46 ± 8.82 | -22.2 ± 7.96 | -16.1 ± 10.9 |
| Age at first experiment of HFA 24-2 (year) | 38.1 ± 9.1 | 36.6 ± 8.6 | 41.0 ± 8.2 | 29.0 ± 11.7 |
| Central corneal thickness (µm) | 537 ± 40 | 530 ± 32 | NA | 534 ± 32 |
| Axial length (mm) | 24.6 ± 1.6 | 26.2 ± 1.6 | 25.0 ± 1.2 | 25.8 ± 2.1 |
| MD of HFA 24-2 at final visit (dB) | -14.3 ± 10.5 | -10.4 ± 9.57 | -25.1 ± 6.75 | -16.6 ± 11.0 |
| Rate of change in HFA 24-2 (dB/year) | -0.49 ± 1.20 | -0.45 ± 2.95 | -0.94 ± 2.51 | -0.06 ± 0.95 |
| Experiment times of HFA 24-2 | 7.14 ± 6.3 | 6.24 ± 5.79 | 3.75 ± 1.39 | 5.04 ± 4.31 |
| Best corrected visual acuity (logMAR) | 0.095 ± 0.44 | -0.046 ± 0.26 | 0.66 ± 0.45 | 0.23 ± 0.54 |

Values are shown as mean ± standard deviation.

**Supplementary Table S2** Comparison of clinical characteristics of the patients with secondary glaucoma between the groups divided by the cut-off value of axial length determined by Youden’s index in the current study

|  | AL<27.7mm | AL>=27.7mm | P value | AL<25.5mm | AL>=25.5mm | P value |
| --- | --- | --- | --- | --- | --- | --- |
| Patients | 101 | 19 |  | 60 | 64 |  |
| Eyes | 165 | 28 |  | 92 | 101 |  |
| Gender (female / male) | **49 / 52** | **4 / 15** | **0.04** | 29 / 31 | 25 / 39 | 0.37 |
| Intraocular pressure at first visit (mmHg) | 19.6 ± 10.4 | 18.9 ± 7.9 | 0.73 | 18.4 ± 8.7 | 20.5 ± 11.2 | 0.16 |
| Spherical equivalent refraction at first visit (D) | **-4.02 ± 5.08** | **-8.7 ± 2.72** | **<0.001** | **-1.48 ± 4.42** | **-7.64 ± 3.67** | **<0.001** |
| MD of HFA 10-2 at first visit (dB) | -18.5 ± 10.3 | -19.6 ± 10.5 | 0.81 | -21.4 ± 10.3 | -16.9 ± 9.86 | 0.13 |
| Age at first experiment of HFA 10-2 (year) | 37.9 ± 9.5 | 34.6 ± 11.9 | 0.44 | **41.8 ± 10.5** | **34.5 ± 8.4** | **0.014** |
| MD of HFA 24-2 at first visit (dB) | -12.00 ± 10.30 | -11.80 ± 9.74 | 0.94 | -10.7 ± 9.38 | -13.10 ± 10.80 | 0.15 |
| Age at first experiment of HFA 24-2 (year) | 34.9 ± 10.3 | 34.3 ± 8.3 | 0.81 | 36.3 ± 11.6 | 33.5 ± 8.3 | 0.13 |
| Central corneal thickness (µm) | 532 ± 39 | 519 ± 28 | 0.14 | 529 ± 43 | 530 ± 34 | 0.88 |
| Axial length (mm) | **25.2 ± 1.5** | **28.7 ± 0.9** | **<0.001** | **24.1 ± 1.0** | **27.2 ± 1.2** | **<0.001** |
| MD of HFA 24-2 at final visit (dB) | -13.6 ± 10.8 | -13.8 ± 10.0 | 0.93 | -12.4 ± 10.4 | -14.8 ± 10.8 | 0.20 |
| Rate of change in HFA 24-2 (dB/year) | -0.45 ± 2.60 | -0.35 ± 0.45 | 0.72 | -0.07 ± 1.45 | -0.83 ± 3.12 | 0.10 |
| Experiment times of HFA 24-2 | 5.9 ± 5.3 | 6.6 ± 7.5 | 0.72 | 6.3 ± 5.5 | 5.7 ± 5.8 | 0.60 |
| Best corrected visual acuity (logMAR) | 0.088 ± 0.42 | 0.13 ± 0.53 | 0.70 | 0.12 ± 0.48 | 0.075 ± 0.40 | 0.56 |

Values are shown as mean ± standard deviation.

Statistically significant values are shown in bold.

Comparison was performed using unpaired t test (continuous value) or Fisher’s exact test (categorical value).

MD: mean deviation, HFA: Humphrey field analyzer.
